# Preparation and Characterization of Extruded PLA Films Coated with Polyaniline or Polypyrrole by In Situ Chemical Polymerization

**DOI:** 10.1021/acsomega.3c07201

**Published:** 2023-11-01

**Authors:** José Ramón Flores León, Jesús Manuel Quiroz Castillo, Dora E. Rodríguez Félix, María Mónica Castillo Ortega, Ana Daymi Cabrera-González, Claudia Georgina Ramirez-Mendoza, Hisila Santacruz-Ortega, Guillermo Suárez-Campos, Jesús Leobardo Valenzuela-García, Pedro Jesús Herrera-Franco

**Affiliations:** † Departamento de Investigación en Polímeros y Materiales, Universidad de Sonora, C.P. 83000 Hermosillo, Sonora, México; ‡ Departamento de Investigación en Física, Universidad de Sonora, C.P. 83000 Hermosillo, Sonora, México; § Departamento de Ingeniería Química y Metalurgia, Universidad de Sonora, C.P. 83000 Hermosillo, Sonora, México; ∥ Unidad de Materiales, Centro de Investigación Científica de Yucatán, C.P. 97205 Mérida, Yucatán, México

## Abstract

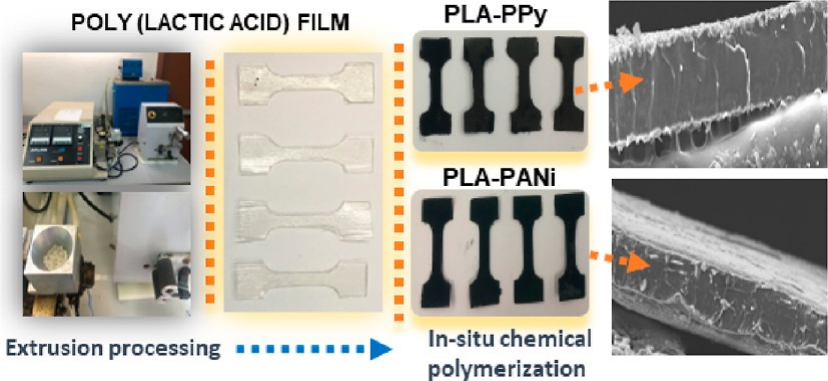

Conductive polymers, such as polypyrrole and polyaniline, have been extensively studied for their notable intrinsic electronic and ionic conductivities, rendering them suitable for a range of diverse applications. In this study, in situ chemical polymerization was employed to coat extruded PLA films with PPy and PANi. Morphological analysis reveals a uniform and compact deposition of both polyaniline and polypyrrole after polymerization periods of 3 and 1 h, respectively. Furthermore, the PLA–PANi-3h and PLA–PPy-1h composites exhibited the highest electrical conductivity, with values of 0.042 and 0.022 S cm^–1^, respectively. These findings were in agreement with the XPS results, as the polyaniline-coated film showed a higher proportion of charge carriers compared to the polypyrrole composite. The elastic modulus of the coated films showed an increase compared with that of pure PLA films. Additionally, the inflection temperatures for the PLA–PANi-3h and PLA–PPy-1h composites were 368.7 and 367.2 °C, respectively, while for pure PLA, it reached 341.47 °C. This improvement in mechanical and thermal properties revealed the effective interfacial adhesion between the PLA matrix and the conducting polymer. Therefore, this work demonstrates that coating biopolymeric matrices with PANi or PPy enables the production of functional and environmentally friendly conductive materials suitable for potential use in the removal of heavy metals in water treatment.

## 1 Introduction

Intrinsically conducting polymers (ICPs) with conjugated π-electron systems, such as polyaniline (PANi), polythiophene, and polypyrrole (PPy), have been extensively researched in recent decades.^1^ PANi and PPy have garnered significant attention due to their ease of polymerization, high intrinsic conductivity (ranging from 0.01 to 500 S cm^–1^ in the doped state), redox reversibility, and environmental stability.^2^ The exceptional properties of these polymers have made them suitable for a wide range of applications, including photovoltaic cells, organic light-emitting diodes, and biosensors.^3−6^ In addition, owing to their excellent ion exchange behavior, considerable attention has been given in recent years to the capacity for removing toxic heavy metal ions such as mercury, nickel, cadmium, and chromium.^7,8^ Nevertheless, their limited surface area, poor mechanical properties, and challenges in conventional processing methods have posed obstacles to fully harnessing their potential.^9,10^ Luckily, the limitations of PANi and PPy can be overcome by creating composite materials that synergistically integrate the desirable mechanical properties of the insulating host matrix with the electrical properties of conductive polymers.^11^ Coating the polymer matrix with ICPs is a promising alternative for obtaining such systems. As a result, researchers have reported the successful deposition of polyaniline or polypyrrole onto polymeric templates using various methods, including in situ chemical oxidative polymerization, spin coating, vapor-phase polymerization, and plasma polymerization.^12−15^ On the other hand, there is growing interest in the use of biopolymeric and/or natural matrix templates. These materials are valuable sources for the advancement of novel materials due to their extensive natural diversity in composition, and biodegradable nature makes them more environmentally friendly in comparison to support matrices derived from synthetic polymers.^16−19^ Several researchers have utilized these biopolymers as matrices in composite materials. In a notable study, El-Nahrawy et al. (2020) successfully created a nanocomposite using cellulose nanowhiskers (CNW) through the in situ emulsion polymerization reaction of pyrrole, with the favorable electrical properties dependent on temperature.^20^ Similarly, Reis et al. (2021) developed a conductive biocomposite based on chitosan and polypyrrole, capable of removing chromium from aqueous solutions.^21^ Finally, Imgharn et al. (2023) achieved the synthesis of a highly porous polyaniline-based hydroxyapatite-montmorillonite composite through in situ chemical polymerization.^22^

Poly(lactic acid) (PLA) is a biopolymer that has garnered significant attention recently due to its remarkable biodegradability, biocompatibility, and exceptional mechanical properties. In addition, it is economically polymerized from lactic acid obtained from corn and sugar beet and can be easily processed using the same techniques as that for conventional polymers.^23^ Picciani et al. (2010) developed conducting electrospun fiber mats based on PLA and PANi. This study achieved homogeneous nanoscale fibers with excellent interfacial adhesion between the electroconductive polymer and the matrix.^24^ Similarly, Leng et al. (2018) prepared core–shell microfibers of PLA–PPy using the electrospinning technique. The results demonstrated the achievement of a highly conductive system with electrical conductivity values of 0.5 S cm^–1^, rendering it suitable for stimulus-responsive applications.^25^ On the other hand, some authors have also reported the preparation of blends based on PLA and conductive polymers. Wong et al. (2020) fabricated a biodegradable material of PLA–PANi through ex situ polymerization using the solution casting method. The results revealed a highly compact and porous material with properties suitable for antistatic packaging applications.^26^ Likewise, Monleón Pradas et al. (2020) fabricated PLA cast membranes coated with polypyrrole; the findings unveiled a uniform and continuous PPy coating, accompanied by a high electrical conductivity, making them candidates for applications such as solid polymer electrolytes.^27^ Although both electrospinning and casting techniques are among the most reported methods for the preparation of PLA–PPy and PLA–PANi systems, to date, there are no reports in the literature of systems based on extruded PLA films coated with PANi or PPy. Therefore, the objective of our work was to develop a viable approach to prepare electroconductive films based on PLA using simple, economically feasible methods and, above all, scalable to an industrial level, such as extrusion molding.

In this regard, in the current work, the coating of extruded PLA films with PANi or PPy was carried out through an in situ chemical oxidation method. The morphological, mechanical, and thermal properties of the films were studied. Additionally, the conductive nature of the materials was demonstrated by assessing their electrical properties, and the chemical composition of the coated films was obtained by FT-IR and XPS analyses.

## 2 Experimental Methods

### 2.1 Materials

Poly(lactic acid) (PLA, 2002d, of 192.61 kDa) for extrusion was obtained from NatureWorks (Blair, NE, USA), and distilled water (H_2_O, analytical purity) and hydrochloric acid (HCl) ACS reagent 37.20% were obtained from Sigma-Aldrich. Aniline (C_6_H_7_N) 99.5% and pyrrole (C_4_H_5_N) 98% (both reagent grade) were purchased from Sigma-Aldrich and were purified by vacuum distillation and stored under refrigeration before use. Hexahydrate ferric chloride (FeCl_3_·6H_2_O) ACS reagent from Fermont and ammonium persulfate (N_2_H_8_S_2_O_8_) ACS reagent 98% were purchased from Sigma-Aldrich, and both were used as oxidizing agents.

### 2.2 Preparation of PLA Films Coated with PANI or PPy by In Situ Chemical Polymerization

The PLA films were prepared by an extrusion process. PLA granules, previously dried at 50 °C for 2 h, were extruded using an Atlas laboratory mixer-extruder, operated at a speed of 35 rpm, and the temperatures were controlled at 160 °C for the screw barrel and 170 °C for the flat die.

For the coating of PLA films with polypyrrole (PLA–PPy), a solution of 0.5 M pyrrole in HCl (1 M) was prepared. The PLA films were cut into strips (1.5 × 1 cm) to facilitate their coating and were immersed in 5 mL of pyrrole solution for 15 min. Subsequently, to start the polymerization reaction, 5 mL of 0.5 M hexahydrate ferric chloride was added under magnetic stirring at 200 rpm, and five polymerization times were studied corresponding to 0.5, 1, 2, 3, and 4 h. For the coating of the films with polyaniline (PLA–PANi), a similar procedure described for the coating with polypyrrole was used, but 0.5 M ammonium persulfate and 0.5 M aniline solutions with equal contact time were used instead. The coated films were washed with deionized water and dried at 25 °C for 24 h.

### 2.3 Characterization

#### 2.3.1 Morphological Analysis

The morphology of coated films was examined using a JEOL JSM-5410LV scanning electron microscope coupled with an energy-dispersive X-ray spectroscope, operated at a voltage of 20 kV. The samples were gold-sputtered before the SEM examination.

#### 2.3.2 Electrical Properties

The electrical conductivity was determined from the I–V curves of the materials and was carried out by using the two-point method. Measurements were made by using a Keithley 2400 A Tektronix Company semiconductor characterization system at room temperature. The measurements were performed under a linear sweep mode from −5 to 5 V and a 2.4 mm separation between the tips.

#### 2.3.3 Fourier Transform Infrared Spectroscopy Analysis

Fourier transform infrared (FTIR) spectra of the samples were obtained with a Thermo Scientific Nicolet iS50 spectrophotometer. The spectra were recorded employing the attenuated total reflectance (ATR) technique, scanning from 4000 to 400 cm ^–1^. An average of 32 scans were recorded.

#### 2.3.4 Tensile Strength Test

The mechanical properties of the films were determined by using a United SSTM-5kN universal testing machine equipped with a 5 kN load cell. A speed of testing of 1 mm/min and a distance between the ends of the gripping surfaces of 20 mm were used. The thickness of the films was determined with a Mitutoyo micrometer, and the dimensions were maintained in the range of 0.3–0.6 mm thickness and a width of 5.2 mm. The samples were conditioned to room temperature and humidity before and during testing. An average of 10 specimens for each test is reported.

#### 2.3.5 Thermogravimetric Analysis

To analyze the coated film samples’ thermal stability, TGA was performed using a Pyris 1 instrument from PerkinElmer. About 4 mg of the materials was placed in a porcelain sample holder; the samples were subject to a temperature increase rate of 10 °C/min from room temperature to 600 °C under a nitrogen atmosphere.

#### 2.3.6 Differential Scanning Calorimetry

DSC measurements were carried out on a PerkinElmer DSC 8500 equipment; samples of 7 mg each were placed in high-purity aluminum sample holders. The temperature was raised from 25 to 200 °C at a rate of 10 °C min ^–1^ under a nitrogen atmosphere. After cooling, the samples were heated again by employing the same heating rate.

#### 2.3.7 XPS Analysis

XPS spectra were measured on a PerkinElmer vacuum Products model PHI 5100 photoelectron spectrometer with Mg Kα exciting radiation at 15 kV and 10 mA; the base pressure was approximately 10–9 Torr. The survey scans were in the range of 0–1100 eV. To compensate for the surface charging effects, all binding energies were referenced to the C (1s) peak at 284.6 eV. A Gaussian deconvolution curve fitting on a Shirley background was performed to evaluate the different contributions associated with the various types of nitrogen (N 1s) and (Cl 2p) bonds present on the material.

## 3 Results and Discussion

### 3.1 SEM Image Analysis

**Figure 1:**
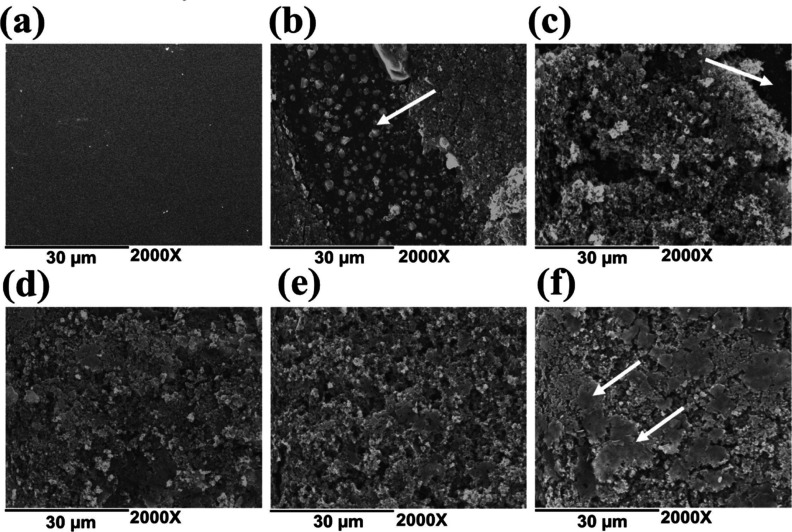
SEM micrographs of polyaniline-coated films at different polymerization times: (a) pure PLA; (b) 0.5; (c) 1; (d) 2; (e) 3; and (f) 4 h at 2000× magnification.

Figure 1 shows the SEM micrographs of PLA films coated with polyaniline (PLA–PANi) at different polymerization times. At the polymerization times of 0.5 and 1 h, as depicted in Figure 1b,c, respectively, the coating showed limited effectiveness, as polyaniline did not fully cover the PLA film surface, leaving significant areas without the conductive polymer. Possibly, the short reaction time did not allow the polymerization of sufficient aniline monomers, resulting in the deposition of a small amount of polyaniline particles.^28,29^ As the reaction time increased, a greater amount of polyaniline was deposited on the surface of the PLA film, resulting in a complete coating at 3 h of polymerization (Figure 1e). In this coating, the PANi particles were uniformly distributed and exhibited a high degree of compaction. For the final polymerization time, corresponding to the 4 h reaction (Figure 1f), agglomeration of the electroconductive polymer and a nonhomogeneous coating on the surface of pure PLA were observed. These findings suggested that a prolonged polymerization time hindered the attainment of a homogeneous film.

**Figure 2:**
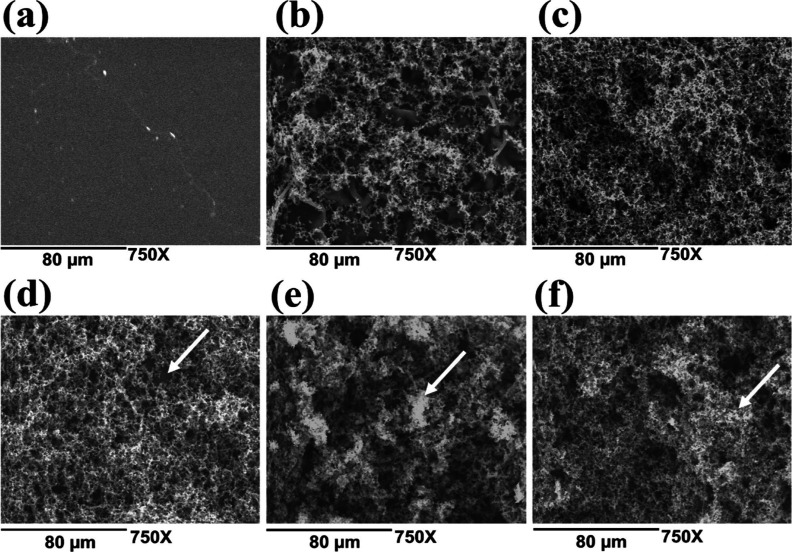
SEM micrographs of polypyrrole-coated films at different polymerization times: (a) pure PLA; (b) 0.5; (c) 1; (d) 2; (e) 3, and (f) 4 h at 750× magnification.

**Figure 3:**
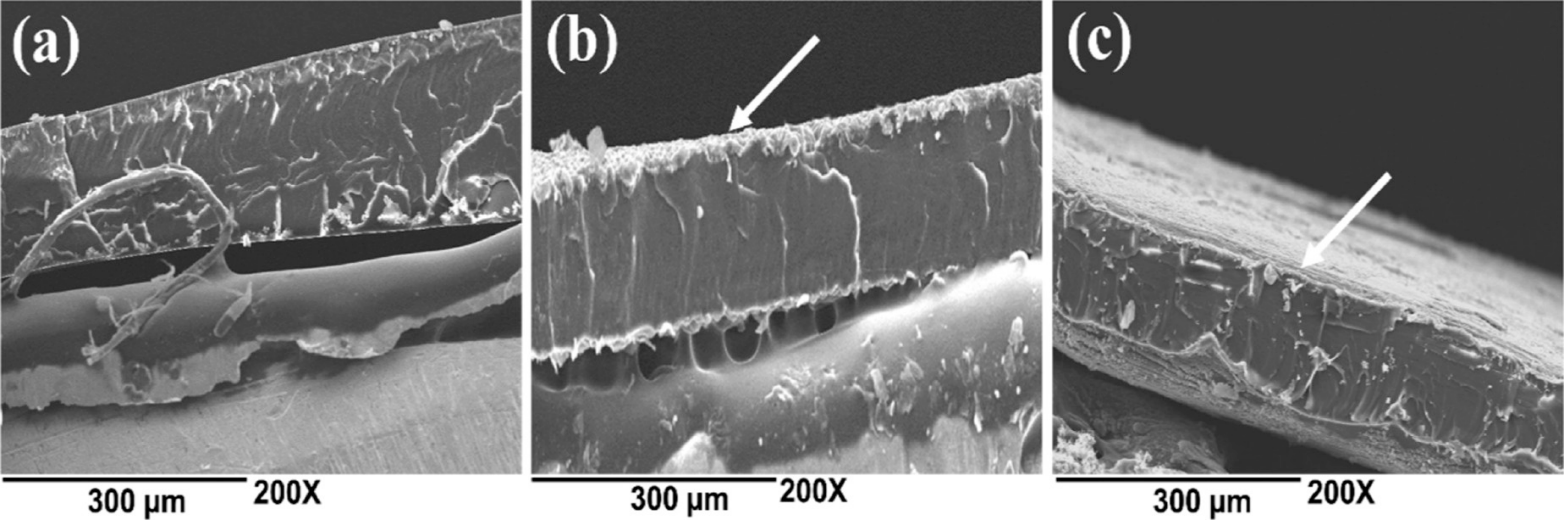
Cross-sectional micrographs of PLA and coated films: (a) PLA; (b) PLA–PPy, and (c) PLA–PANi at 200× magnification.

Figure 2 presents the SEM micrographs of PLA films coated with polypyrrole (PLA–PPy) at different polymerization times. Unlike the films coated with polyaniline, 1 h of polymerization was sufficient for polypyrrole to uniformly coat the PLA film (Figure 2c). This can be observed more clearly in the cross-sectional SEM micrographs (Figure 3b), where a thin, homogeneous layer of the electroconductive polymer is distributed on the surface of the PLA matrix. Additionally, starting from 2 h of reaction (Figure 2d), agglomeration of the electroconductive polymer was observed. This agglomeration progressively increased as the reaction time was extended. This behavior became more pronounced after 4 h of polymerization (Figure 2f).

**Figure 4:**
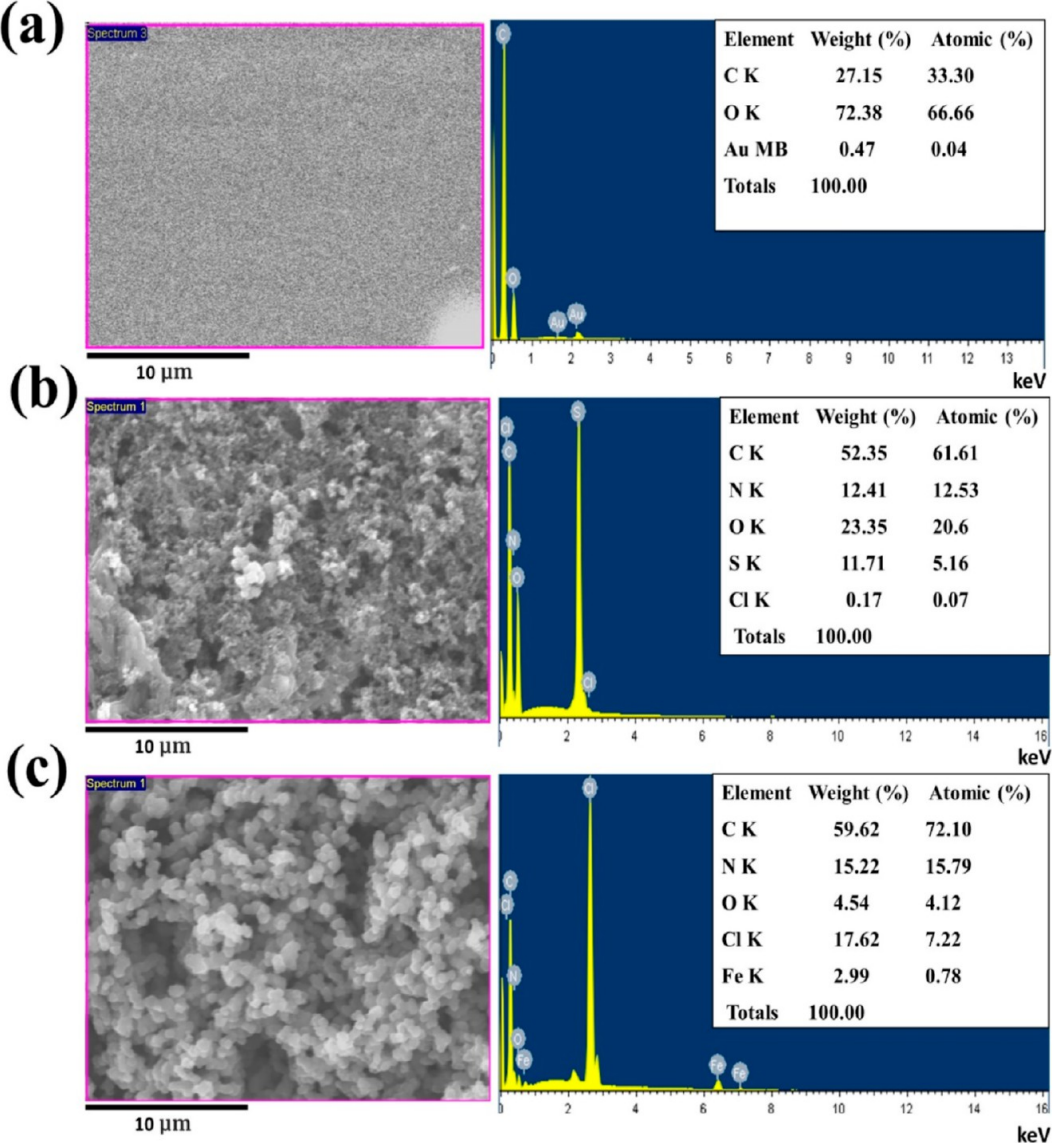
SEM–EDX analysis of the films: (a) neat PLA, (b) PLA–PANi-3h, and (c) PLA–PPy-1h at 5000× magnification.

The SEM–EDX analysis of neat PLA films (a) and coated PLA films with PANi at 3 h of polymerization (b) and PPy at 1 h of polymerization (c) is depicted in Figure 4. The EDX analysis of the neat PLA film reveals the presence of carbon (C) and oxygen (O) atoms, along with gold (Au), which is a result of the prior coating before analysis. Furthermore, both coated films show discernible peaks corresponding to C and N, confirming the incorporation of PANi and PPy particles onto the PLA film.^30^ Finally, sulfur and iron are observed for the polyaniline-coated and polypyrrole-coated films, respectively. This suggests the potential existence of residues from the oxidizing agents used in the reaction.^31^

### 3.2 Electrical Properties

Polypyrrole and polyaniline are among the most studied conductive polymers due to their long π-conjugated length, unique electrical properties, reversible doping/dedoping process, and controllable chemical and electrochemical properties. When these electroactive polymers are homogeneously deposited on the surface of a film, their electroconductive capacity can be transferred to the composite material and even enhanced.^13,32^

**Figure 5:**
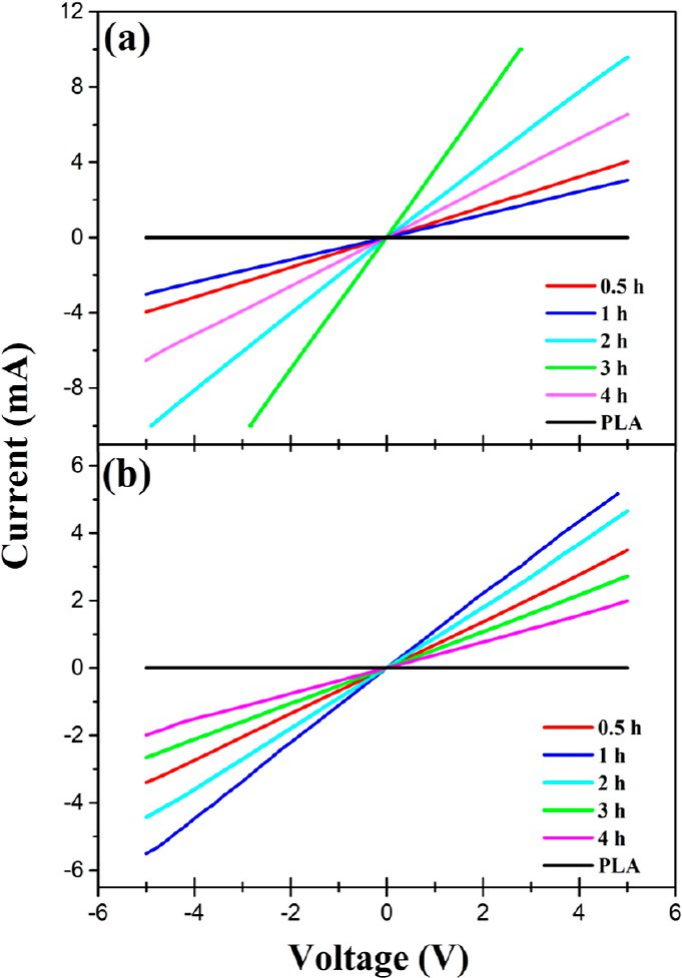
Current–voltage plots of coated films at different polymerization times. (a) PLA–PANi and (b) PLA–PPy.

In Figure 5a,b, the I–V curves of PLA–PANi and PLA–PPy, respectively, at different polymerization times are illustrated. It is evident that the pristine PLA film exhibits an insulating behavior with no measurable conductivity. On the contrary, from the initial 0.5 h of in situ polymerization of pyrrole and aniline, the composite films became electrically conductive. Specifically, for the PLA–PANi film, the electrical conductivity exhibited an increase as the reaction time prolonged (Figure 5a). With the increase in the reaction time, a higher number of polyaniline particles were deposited onto the PLA film, facilitating electron transfer within the composite film and resulting in a gradual increase in electrical conductivity.^33^ In particular, at 3 h of polymerization, the PLA–PANi film presented the highest electrical conductivity with a value of 0.042 S cm^–1^ (Table 1), which is attributed to the highly compact and homogeneous distribution of PANi particles observed through scanning electron microscopy (Figure 1e).^34^ Additionally, at 4 h of polymerization, the PLA–PANi film presented a decrease in electrical conductivity as it exhibited a conductivity of 0.0172 S cm^–1^. This behavior could be related to the overoxidation of the PANi chains.^34,35^

**Table 1:** Electrical Conductivity of Films Coated After Different Polymerization Times

	Electrical conductivity (S cm–1)	
Polymerization time (h)	PLA–PPy	PLA–PANi
0.5	0.0090 ± 1.50 × 10^–^^4^	0.0091 ± 3.30 × 10^–^^3^
1	0.0220 ± 1.10 × 10^–^^3^	0.0066 ± 8.48 × 10^–^^3^
2	0.0107 ± 3.05 × 10^–^^4^	0.0220 ± 6.61 × 10^–^^3^
3	0.0073 ± 3.24 × 10^–^^4^	0.0420 ± 7.10 × 10^–^^3^
4	0.0075 ± 1.30 × 10^–^^3^	0.0172 ± 4.47 × 10^–^^3^

On the other hand, the PLA–PPy film showed its highest electrical conductivity during the first hour of polymerization, with a value of 0.0220 S cm^–1^. Similarly, for the PLA–PANi film, this was attributed to the high compaction of the polypyrrole particles and their homogeneous distribution (Figure 2c), which facilitates proper charge percolation.^36,37^ After the first hour of polymerization, the electrical conductivity of the PLA–PPy films gradually decreased (Figure 5b). This could be related to phase separation present in the composite film due to the formation of polypyrrole agglomerates on the surface of the PLA film.^38,39^

### 3.3 FTIR Spectroscopy Analysis

**Figure 6:**
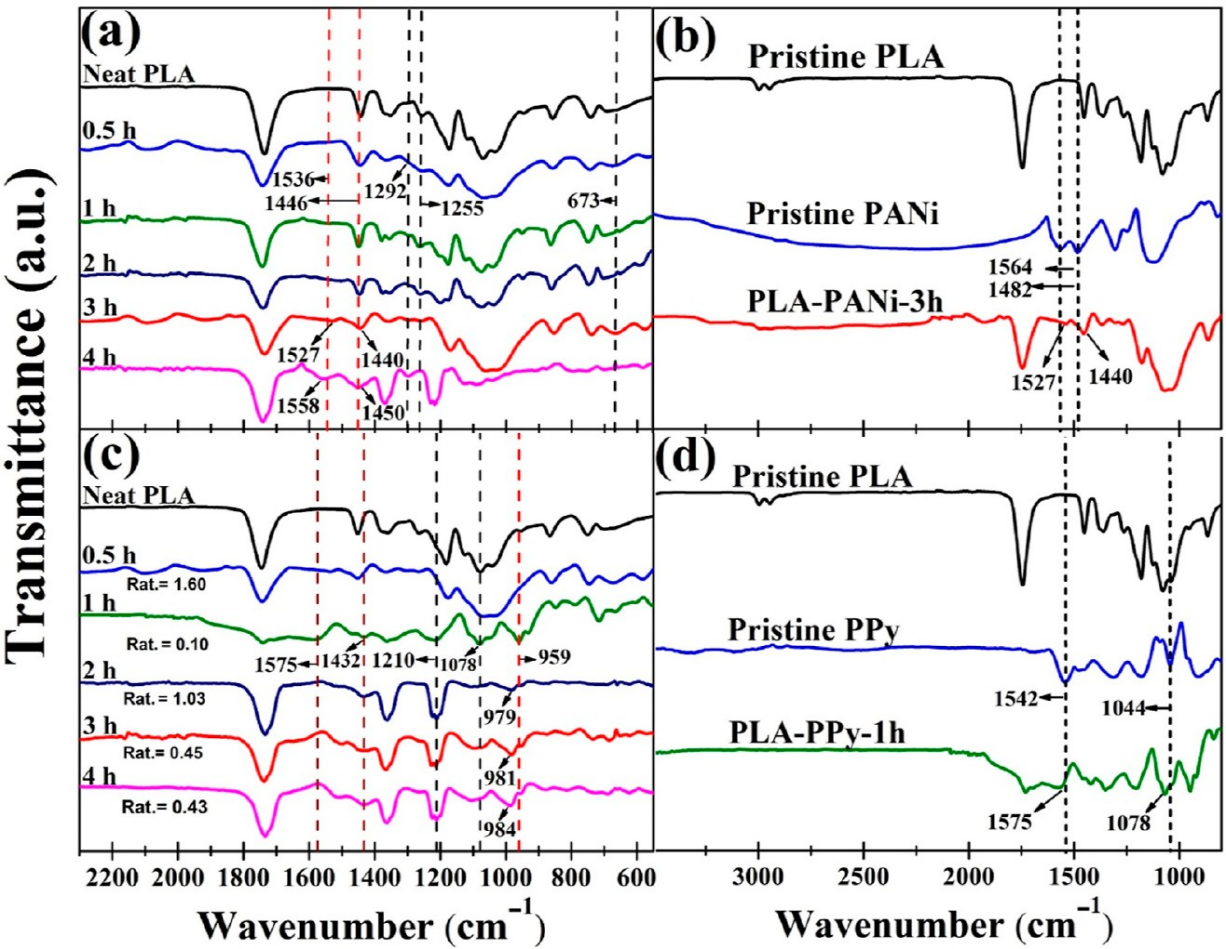
FTIR spectra of coated films obtained at different polymerization times: (a) PANi-coated and (c) PPy-coated films, respectively. Subfigures (b,d) correspond to pristine polyaniline and polypyrrole, as well as their respective composite films.

Figure 6a shows the FT-IR spectra of the PLA–PANi films at different polymerization times. For each reaction time, the characteristic bands of PANi are present, thereby confirming the presence of polyaniline on the PLA film. The main peaks of polyaniline were located around 1536, 1446, 1292, and 1255 cm^–1^, which were characteristic of the –C=N quinoid ring, –C=C– benzenoid structure, C–N^+^ protonated aromatic amine, and C–N deprotonated amine.^40,41^ Additionally, the peak at 673 cm^–1^ was assigned to the out-of-plane bending vibration of the symmetric =C–H bonds in the benzene ring.^42^ The intensity and position of the peaks associated with the quinoid and benzenoid rings are strongly related to the conducting states of polyaniline in the coated films. It can be observed that the position of these peaks shifts from 1536 to 1527 cm^–1^ and from 1446 to 1440 cm^–1^, respectively, as the polymerization time increases from 0.5 to 3 h. This shift is accompanied by an increase in the intensity of the 1527 cm^–1^ band and a decrease in the intensity of the 1440 cm^–1^ band (Table 2). These results suggest the formation of highly oxidized protonated states that can be attributed to the improvement in the conjugation and conductivity states of PANi with the increasing polymerization time.^33^ For the film obtained at 4 h of polymerization, the position of these bands shifted to a higher wavenumber, and the intensity of the 1450 cm^–1^ band increased, which may be attributed to the overoxidation of the coated film.^33,43^ These results are consistent with the electrical conductivity analysis (Table 1). However, variations were observed in the FTIR spectra of the PLA–PANi coated films compared with pure PANi. Specifically, in the PLA–PANi-3h film (Figure 6b), it was noticed that the peaks originally located at 1564 and 1482 cm^–1^ shifted to 1527 and 1440 cm^–1^, respectively. These changes suggest an interaction between polyaniline and the polymeric structure of PLA, possibly due to the formation of hydrogen bonds.^44,45^

**Table 2:** Changes in the Absorption Bands in the Spectra of PLA–PANi Films at Different Polymerization Times

Vibration mode	Polymerization time (h)	Integration area band	Absorption peak wavenumbers (cm^–1^)
–C=N stretching of quinoid ring	0.5	8.13	1536
	1	46.38	1538
	2	41.22	1536
	3	144.35	1527
	4	340.01	1558
–C=C– stretching of benzenoid rings	0.5	1037.20	1446
	1	790.51	1447
	2	475.61	1446
	3	402.15	1440
	4	731.12	1450

The FT-IR spectra of the PLA–PPy films at different polymerization times are presented in Figure 6c. For each polymerization time, the main peaks of polypyrrole are displayed. The peaks at 1575 and 1432 cm^–1^ are attributed to the symmetric and antisymmetric vibrations of C=C–N in the plane of the benzene ring, and peaks at 1210 and 1078 cm^–1^ correspond to the C–N in-plane deformation and the vibration of the N–H bond, respectively.^46,47^ The peak observed at 959 cm^–1^ corresponds to the stretching vibration of C=N^+^–C. This particular peak at 959 cm^–1^ confirms that PPy was effectively doped with FeCl_3_, resulting in positively charged units that act as charge carriers, i.e., polarons/bipolarons.^48,49^ It is worth noting that the position of this peak shifts to higher wavenumbers as the polymerization time increases from 1 to 4 h. These findings suggest that reducing the polymerization time results in an increased electron density induced by charge transfer within the coated film.^50^ To complement these findings, we determined the effective “conjugation length” of PPy in the films. The degree of electron delocalization is directly proportional to the ratio of peak areas at 1432 and 1575 cm^–1^, and conductivity correlates directly with the *A*_1432_/*A*_1575_ ratio.^51^ In general, a decrease in this ratio was observed as the polymerization time increased. Specifically, the film obtained at 1 h exhibited the lowest value of 0.10. These findings align with the electrical conductivity values as the PLA–PPy-1h film proved to be the most conductive. Finally, the peaks initially observed at 1542 and 1044 cm^–1^ for pristine PPy shifted to 1575 and 1078 cm^–1^ for the PLA–PPy-1h films Figure 6d, indicating the intermolecular interaction between the PLA matrix and polypyrrole.^46^

### 3.4 Mechanical Properties

**Figure 7:**
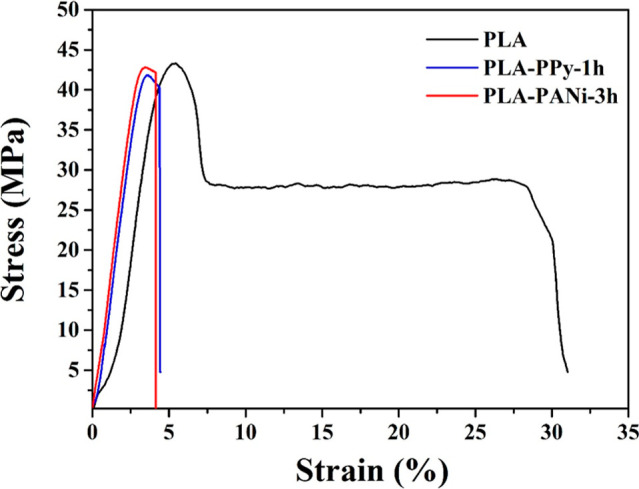
Stress–strain curves of pure PLA, PLA–PPy-1h, and PLA–PANi-3h.

The stress–strain curves of the pristine films and the films coated with electroconductive polymers are depicted in Figure 7. As observed, the pure PLA film exhibited a tensile strength of 44.27 MPa and an elongation at break of 26.48%. Furthermore, the modulus of elasticity for this film was 1075.39 MPa. These findings are consistent with those reported by several authors in the literature.^52,53^ Nevertheless, the addition of electroconductive polymers to the PLA matrix resulted in a series of variations in the material’s properties. First, a slight decrease in tensile strength was observed for both composite materials, presenting values of 43.20 and 43.90 MPa for the PLA–PPy-1h and PLA–PANi-3h films, respectively. This decrease is attributed to the brittle nature of the aromatic rings present in polypyrrole and polyaniline.^54,55^ Contrarily, the composite films presented an increase in their modulus of elasticity as compared to the pure PLA film. This behavior became more evident in the film coated with polyaniline (PLA–PANi-3h), since it presented Young’s modulus of 1609.63 MPa. The increase is associated with a decrease in the flexibility of the PLA polymer chains because of their intermolecular interaction with polypyrrole and polyaniline.^56,57^ Finally, the elongation values of the PLA–PPy-1h and PLA–PANi-3h films showed a reduction of approximately 80% compared to that of neat PLA, confirming the increase in rigidity upon the incorporation of PPy and PANi particles.^58^

### 3.5 Thermal Properties

**Figure 8:**
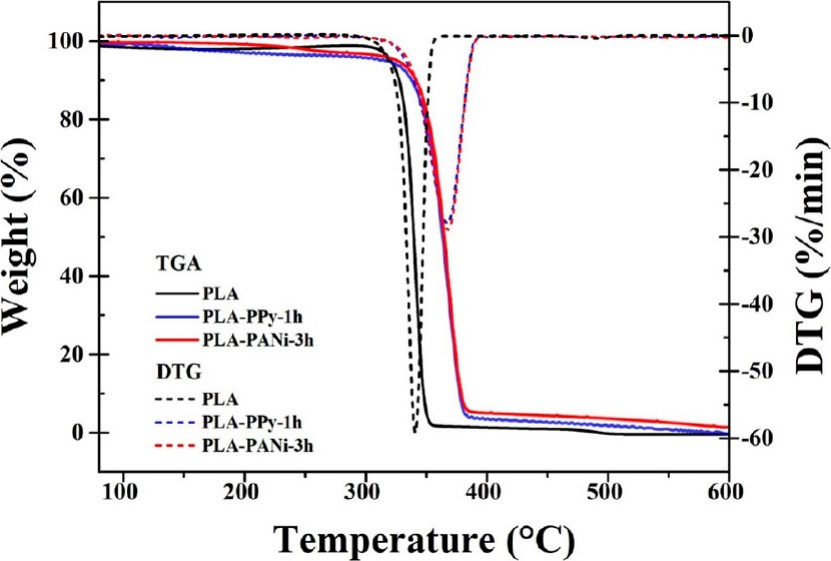
TGA and DTG curves of PLA, PLA–PPy-1h, and PLA–PANi-3h films.

TGA and derivative thermogravimetry (DTG) curves of neat PLA, PLA–PPy-1h, and PLA–PANi-3h films are presented in Figure 8. The neat PLA film shows a single degradation step, characterized by a weight loss onset at approximately 308.42 °C, corresponding to the degradation of polyester chains. Additionally, the film exhibits an inflection temperature (*T*_max_) of 341.47 °C (as confirmed by the DTG signal). These results are in agreement with previous literature reports.^59−61^ On the other hand, the coated films exhibited higher thermal stability compared to the pure PLA films, as evidenced by their higher inflection temperatures of 367.2 and 368.7 °C for the PLA–PPy-1h and PLA–PANi-3h films, respectively. This enhancement in thermal stability can be attributed to the effective interfacial adhesion between the PLA and the electroconductive polymers.^62,63^ Finally, the coated films, like the pure PLA film, demonstrated degradation in a single step, indicating a continuous and homogeneous PPy and PANi coating on the PLA films.^64^

**Figure 9:**
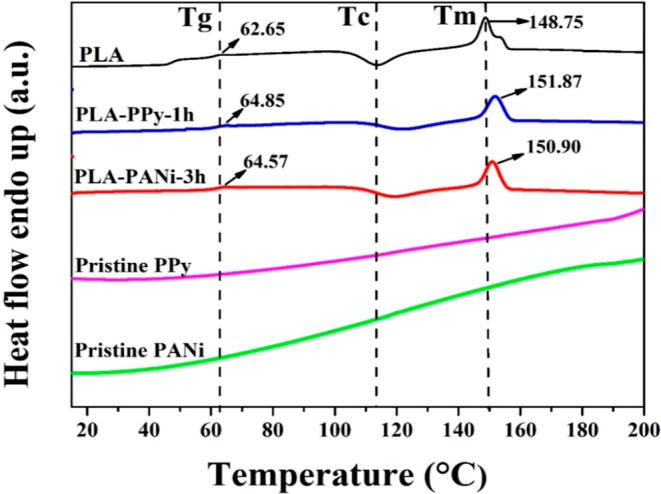
DSC thermograms of neat components, PLA–PPy-1h, and PLA–PANi-3h.

The DSC curves up to 200 °C obtained for pure PLA, PLA–PPy-1h, and PLA–PANi-3h are presented in Figure 9. It is evident that the incorporation of PANi and PPy increases the thermal properties of the composite films. Regarding neat PLA, the glass-transition temperature (*T*_g_) was observed at 62.65 °C, while for the composites, it increased slightly to 64.85 and 64.57 °C for PLA–PPy-1h and PLA–PANi-3h, respectively. This shift in *T*_g_ can be attributed to the intermolecular interactions between PLA and conductive polymers, as observed by FT-IR analysis.^65,66^ Similarly, the addition of PANi and PPy increases the crystallization temperature (*T*_c_) and the melting temperature (*T*_m_). Specifically, the melting peaks are found at 151.87 and 150.90 °C for PLA–PPy-1h and PLA–PANi-3h, respectively, while for neat PLA, it was at 148.75 °C. This enhancement in temperature values is attributed to the nucleating action of the PPy and PANi particles onto the PLA matrix.^67,68^ Finally, the degree of crystallinity (*X*_c_) of the neat PLA and coated films was calculated by the following equation^69^
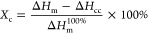
1where Δ*H*_m_ refers to the melting enthalpy of the films, Δ*H*_cc_ represents crystallization enthalpy, and Δ*H*_m_^100%^ represents the melting enthalpy of 100% crystalline PLA (93.6 J/g). As indicated in Table 3, the calculated *X*_c_ values were 42.04, 28.64, and 33.62% for neat PLA, PLA–PPy-1h, and PLA–PANi-3h, respectively. These results are consistent with the obtained melting enthalpies (Δ*H*_m_), showing that the enthalpies of the coated films decrease compared with those of pure PLA.^70^

**Table 3:** Crystallinity Percentages of the Pure and Coated Films

ID	Δ*H*_m_ (J/g)	Δ*H*_cc_ (J/g)	*X*_c_ (%)
PLA	24.936	–14.42	42.04
PLA–PPy-1h	17.43	–9.38	28.64
PLA–PANi-3h	19.10	–12.37	33.62

### 3.6 XPS Analysis

**Figure 10:**
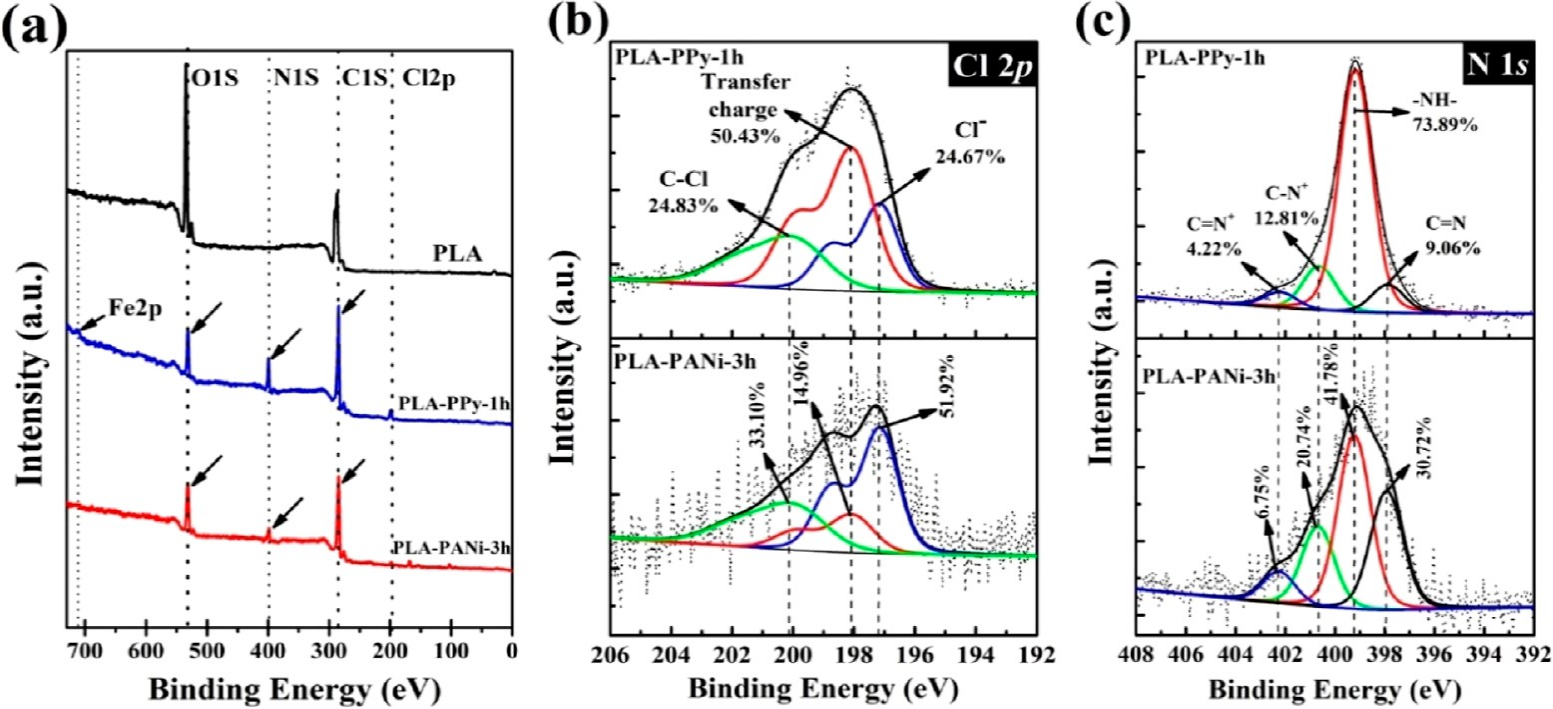
XPS spectra: (a) wide-scan survey of PLA, PLA–PPY-1h, and PLA–PANi-3h; and high-resolution spectra from Cl 2p (b) and N 1s (c) core levels.

The XPS patterns were employed to validate the elemental composition and chemical state of the samples. Figure 10a shows the XPS survey spectrum of the obtained films. Comparison with PLA, PLA–PPy-1h, and PLA–PANi-3h films exhibits an additional peak corresponding to N 1s, indicating the successful coating of PPy and PANi, respectively.^71^ Additionally, both coated films exhibited binding energy peaks at approximately 284.50 and 531.50 eV, which were attributed to C 1s and O 1s, respectively.^72^ Furthermore, the PLA–PPy-1h film exhibited a peak related to Fe 2p located at 710.10 eV, possibly corresponding to ferric chloride residues present in the coated film.^73^ Moreover, the high-resolution spectra of Cl 2p presented in Figure 10b revealed that Cl in PLA–PPy and PLA–PANi existed primarily in three forms: chloride anion (Cl^–^) located at 197.14 eV, (Cl^–^) resulting from the charge transfer with PPy and PANi polarons at the binding energy of 198.10 eV, and covalently bonded chlorine located at 200.11 eV.^74,75^

The deconvoluted N 1s spectra of PLA–PPY-1h and PLA–PANi-3h composite films are presented in Figure 10c. The deconvoluted N 1s spectrum of the coated films showed four peaks located at 397.88, 399.18, 400.63, and 402.23 eV. The peaks at 397.88 and 399.18 eV could be attributed to the nitrogen atoms in the amine (−NH−) and imine (–C=N) structures, respectively, while the peaks at 400.63 and 402.23 eV confirm the presence of the polaron (C–N^+^) and the bipolaron (C=N^+^) structures, respectively.^75^ Furthermore, when comparing the different amounts of N^+^ in the coated films, it was observed that in the PLA–PANi-3h film, the proportion of N^+^ (27.49%) was higher compared to that in PLA–PPy-1h (17.03%). This finding was coherent with the doping level (N^+^/N) of the films, as the polyaniline-coated film exhibited a value of 0.27, while the polypyrrole-coated film had a value of 0.17.^75,76^ These results were consistent with the higher conductivity of the polyaniline composite compared with that of polypyrrole (Table 1).

## 4 Conclusions

Novel conductive biomaterials were obtained by coating extruded PLA films with polyaniline and polypyrrole. FT-IR and EDS analyses confirmed the successful incorporation of PANi and PPy particles onto the PLA film surface. The composites obtained after 3 and 1 h of polymerization for PANi and PPy, respectively, exhibited a homogeneous coating. The PLA–PANi-3h composite exhibited higher electrical conductivity compared to PLA–PPy-1h, which is attributed to the greater percentage of charge carriers (polarons and bipolarons) in the polyaniline-based film, as revealed by XPS. The addition of PANi and PPy improved the thermal stability of the material. Additionally, the composite films showed an increase in glass-transition temperatures (*T*_g_) and fusion temperatures (*T*_m_) compared to neat PLA. These findings are attributed to the effective interfacial adhesion between the conductive polymers and polylactic acid (PLA). Finally, the incorporation of conductive particles increased the elastic modulus of the composite material. In conclusion, the appropriate physicochemical properties and electroactive behavior of these conductive films make them suitable for potential use in the removal of heavy metals in water treatment.

## References

[ref1] Thadathil A.; Pradeep H.; Joshy D.; Ismail Y. A.; Periyat P. Polyindole and polypyrrole as a sustainable platform for environmental remediation and sensor applications. Environ. Chem. Lett. 2022, 3, 2990–3022. 10.1039/D2MA00022A.

[ref2] Al-Zahrani S. A.; Khan I. Preparation and characterization of CNTs reinforced polyaniline@Zn-CuO nanocomposite for environmental applications. Alexandria Eng. J. 2021, 60 (5), 4857–4864. 10.1016/j.aej.2021.03.044.

[ref3] Talukder M. M.; Rahman Khan M. M.; Amin M. K. A review on polyaniline (PANI) based nanocomposites for water purification. S. Afr. J. Chem. Eng. 2023, 44, 276–282. 10.1016/j.sajce.2023.02.004.

[ref4] Li R.; Tang Q.; Yu L.; Yan X.; Zhang Z.; Yang P. Counter electrodes from conducting polymer intercalated graphene for dye-sensitized solar cells. J. Power Sources 2016, 309, 231–237. 10.1016/j.jpowsour.2016.01.095.

[ref5] Elumalai P.; Charles J. Investigation of structural and optical properties of ternary polyaniline–polypyrrole–nickel oxide (PANI-PPy-NiO) nanocomposite for optoelectronic devices. Polym. Int. 2023, 72, 176–188. 10.1002/pi.6456.

[ref6] Fraser S. A.; van Zyl W. E. *In situ* polymerization and electrical conductivity of polypyrrole/cellulose nanocomposites using Schweizer’s reagent. RSC Adv. 2022, 12, 22031–22043. 10.1039/D2RA04320C. 36043106 PMC9361926

[ref7] Ghorbani M.; Eisazadeh H. Removal of COD, color, anions and heavy metals from cotton textile wastewater by using polyaniline and polypyrrole nanocomposites coated on rice husk ash. Composites, Part B 2013, 45 (1), 1–7. 10.1016/j.compositesb.2012.09.035.

[ref8] Zhou F.; Li Y.; Wang S.; Wu X.; Peng J.; Wang F.; Wang L.; Mao J. Turning waste into valuables: In situ deposition of polypyrrole on the obsolete mask for Cr (VI) removal and desalination. Sep. Purif. Technol. 2023, 306 (Part B), 122643 10.1016/j.seppur.2022.122643. 36406342 PMC9661547

[ref9] Salehi M. H.; Golbaten-Mofrad H.; Jafari S. H.; Goodarzi V.; Entezari M.; Hashemi M.; Zamanlui S. Electrically conductive biocompatible composite aerogel based on nanofibrillated template of bacterial cellulose/polyaniline/nano-clay. Int. J. Biol. Macromol. 2021, 173, 467–480. 10.1016/j.ijbiomac.2021.01.121. 33484804

[ref10] Číková E.; Mičušík M.; Šišková A.; Procházka M.; Fedorko P.; Omastová M. Conducting electrospun polycaprolactone/polypyrrole fibers. Synth. Met. 2018, 235, 80–88. 10.1016/j.synthmet.2017.11.011.

[ref11] Chen D.; Miao Y.-E.; Liu T. Electrically Conductive Polyaniline/Polyimide Nanofiber Membranes Prepared via a Combination of Electrospinning and Subsequent In situ Polymerization Growth. ACS Appl. Mater. Interfaces 2013, 5 (4), 1206–1212. 10.1021/am303292y. 23347585

[ref12] Chen Y.; Han M.; Tang Y.; Bao J.; Li S.; Lan Y.; Dai Z. Polypyrrole–polyoxometalate/reduced graphene oxide ternary nanohybrids for flexible, all-solid-state supercapacitors. Chem. Commun. 2015, 51 (62), 12377–12380. 10.1039/C5CC02717A. 26140676

[ref13] Syed J. A.; Lu H.; Tang S.; Meng X. Enhanced corrosion protective PANI-PAA/PEI multilayer composite coatings for 316SS by spin coating technique. Appl. Surf. Sci. 2015, 325, 160–169. 10.1016/j.apsusc.2014.11.021.

[ref14] Kim J.-Y.; Lee J.-H.; Kwon S. The manufacture and properties of polyaniline nano-films prepared through vapor-phase polymerization. Synth. Met. 2007, 157, 336–342. 10.1016/j.synthmet.2007.03.013.

[ref15] Goktas H.; Demircioglu Z.; Sel K.; Gunes T.; Kaya I. The optical properties of plasma polymerized polyaniline thin films. Thin Solid Films 2013, 548, 81–85. 10.1016/j.tsf.2013.09.013.

[ref16] Basavaraja C.; Kim W. J.; Kim D. G.; Huh D. S. Synthesis and characterization of soluble polypyrrole–poly(ε-caprolactone) polymer blends with improved electrical conductivities. Mater. Chem. Phys. 2011, 129 (3), 787–793. 10.1016/j.matchemphys.2011.05.057.

[ref17] Stetsiv Y. A.; Yatsyshyn M. M.; Nykypanchuk D.; Korniy S. A.; Saldan I.; Reshetnyak O. V.; Bednarchuk T. J. Characterization of polyaniline thin films prepared on polyethylene terephthalate substrate. Polym. Bull. 2021, 78, 6251–6265. 10.1007/s00289-020-03426-7.

[ref18] AL-Oqla F. M.; Sapuan S. M.; Anwer T.; Jawaid M.; Hoque M. E. Natural fiber reinforced conductive polymer composites as functional materials: A review. Synth. Met. 2015, 206, 42–54. 10.1016/j.synthmet.2015.04.014.

[ref19] Ding Y.; Feng W.; Huang D.; Lu B.; Wang P.; Wang G.; Ji J. Compatibilization of immiscible PLA-based biodegradable polymer blends using amphiphilic di-block copolymers. Eur. Polym. J. 2019, 118, 45–52. 10.1016/j.eurpolymj.2019.05.036.

[ref20] El-Nahrawy A. M.; Abou Hammad A. B.; Khattab T. A.; Haroun A.; Kamel S. Development of electrically conductive nanocomposites from cellulose nanowhiskers, polypyrrole and silver nanoparticles assisted with Nickel (III) oxide nanoparticles. React. Funct. Polym. 2020, 149, 104533 10.1016/j.reactfunctpolym.2020.104533.

[ref21] Reis E. d. S.; Gorza F. D. S.; Pedro G. D. C.; Maciel B. G.; da Silva R. J.; Ratkovski G. P.; de Melo C. P. (Maghemite/Chitosan/Polypyrrole) nanocomposites for the efficient removal of Cr (VI) from aqueous media. J. Environ. Chem. Eng. 2021, 9 (1), 104893 10.1016/j.jece.2020.104893.

[ref22] Imgharn A.; Laabd M.; Naciri Y.; Hsini A.; Mahir F.-Z.; Zouggari H.; Albourine A. Insights into the performance and mechanism of PANI@Hydroxapatite-Montmorillonite for hexavalent chromium Cr (VI) detoxification. Surf. Interfaces 2023, 36, 102568 10.1016/j.surfin.2022.102568.

[ref23] Zhou H.; Lawrence J. G.; Bhaduri S. B. Fabrication aspects of PLA-CaP/PLGA-CaP composites for orthopedic applications: A review. Acta Biomater. 2012, 8 (6), 1999–2016. 10.1016/j.actbio.2012.01.031. 22342596

[ref24] Picciani P. H. S.; Medeiros E. S.; Pan Z.; Wood D. F.; Orts W. J.; Mattoso L. H. C.; Soares B. G. Structural, Electrical, Mechanical, and Thermal Properties of Electrospun Poly(lactic acid)/Polyaniline Blend Fibers. Macromol. Mater. Eng. 2010, 295 (7), 618–627. 10.1002/mame.201000019.

[ref25] Zhang F.; Xia Y.; Wang L.; Liu L.; Liu Y.; Leng J. Conductive Shape Memory Microfiber Membranes with Core–Shell Structures and Electroactive Performance. ACS Appl. Mater. Interfaces 2018, 10 (41), 35526–35532. 10.1021/acsami.8b12743. 30248257

[ref26] Wong P.-Y.; Phang S.-W.; Baharum A. Effects of synthesised polyaniline (PAni) contents on the anti-static properties of PAni-based polylactic acid (PLA) films. RSC Adv. 2020, 10, 39693–39699. 10.1039/D0RA07076A. 35515408 PMC9057396

[ref27] Gisbert Roca F.; García-Bernabé A.; Compañ Moreno V.; Martínez-Ramos C.; Monleón Pradas M. Solid Polymer Electrolytes Based on Polylactic Acid Nanofiber Mats Coated with Polypyrrole. Macromol. Mater. Eng. 2021, 306. 10.1002/mame.202000584.

[ref28] Zhang Y.; Pan T.; Yang Z. Flexible polyethylene terephthalate/polyaniline composite paper with bending durability and effective electromagnetic shielding performance. Chem. Eng. J. 2020, 389, 124433 10.1016/j.cej.2020.124433.

[ref29] Zhang Z.; Wang G.; Gu W.; Zhao Y.; Tang S.; Ji G. A breathable and flexible fiber cloth based on cellulose/polyaniline cellular membrane for microwave shielding and absorbing applications. J. Colloid Interface Sci. 2022, 605, 193–203. 10.1016/j.jcis.2021.07.085. 34325341

[ref30] Abdel Aziz A. H.; Jamil T. S.; Shalaby M. S.; Shaban A. M.; Souaya E. R.; Abdel Ghany N. A. Application of (polyaniline/zeolite X) composite as anticorrosion coating for energy recovery devices in RO desalination water plants. Int. J. Ind. Chem. 2019, 10, 175–191. 10.1007/s40090-019-0182-7.

[ref31] Majumdar S.; Mahanta D. Deposition of an ultra-thin polyaniline coating on a TiO2 surface by vapor phase polymerization for electrochemical glucose sensing and photocatalytic degradation. RSC Adv. 2020, 10 (30), 17387–17395. 10.1039/D0RA01571G. 35515627 PMC9053401

[ref32] Kalotra S.; Mehta R. Synthesis of polyaniline/clay nanocomposites by in situ polymerization and its application for the removal of Acid Green 25 dye from wastewater. Polym. Bull. 2021, 78, 2439–2463. 10.1007/s00289-020-03222-3.

[ref33] Sharma A.; Goyal P. K.; Rawal I.; Rajpal A.; Khokhar A.; Kumar V.; Dahiya S. Structural characteristics and opto-electrical properties of in-situ synthesized polyaniline films. Opt. Mater. 2022, 131, 112712 10.1016/j.optmat.2022.112712.

[ref34] Tian M.; Wang Y.; Qu L.; Zhu S.; Han G.; Zhang X.; Zhou Q.; Du M.; Chi S. Electromechanical deformation sensors based on polyurethane/polyaniline electrospinning nanofibrous mats. Synth. Met. 2016, 219, 11–19. 10.1016/j.synthmet.2016.05.005.

[ref35] Merlini C.; Barra G. M. O.; Ramôa S. D. A. S.; Contri G.; Almeida R. D. S.; d’Ávila M. A.; Soares B. G. Electrically conductive polyaniline-coated electrospun poly(vinylidene fluoride) mats. Front. Mater. Sci. 2015, 2, 14 10.3389/fmats.2015.00014.

[ref36] Bertolini M. C.; Ramoa S. D. A. S.; Merlini C.; Barra G. M. O.; Soares B. G.; Pegoretti A. Hybrid composites based on thermoplastic polyurethane with a mixture of carbon nanotubes and carbon black modified with polypyrrole for electromagnetic shielding. Front. Mater. Sci. 2020, 7, 174 10.3389/fmats.2020.00174.

[ref37] Pelíšková M.; Vilčáková J.; Omastová M.; Sáha P.; Li C.; Quadrat O. The effect of pressure deformation on dielectric and conducting properties of silicone rubber/polypyrrole composites in the percolation threshold region. Smart Mater. Struct. 2005, 14 (5), 949–952. 10.1088/0964-1726/14/5/032.

[ref38] Aradhana R.; Mohanty S.; Nayak S. K. Synergistic effect of polypyrrole and reduced graphene oxide on mechanical, electrical and thermal properties of epoxy adhesives. Polymer 2019, 166, 215–228. 10.1016/j.polymer.2019.02.006.

[ref39] Krushnamurty K.; Rini M.; Srikanth I.; Ghosal P.; Das A. P.; Deepa M.; Subrahmanyam Ch. Conducting polymer coated graphene oxide reinforced C-epoxy composites for enhanced electrical conduction. Composites, Part A 2016, 80, 237–243. 10.1016/j.compositesa.2015.10.030.

[ref40] Yu P.; Wu R.; Liu C.; Lan J.; Lin Y.; Yang X. Polyaniline/SWCNT composite films prepared via the solvent-induced strategy for flexible energy harvesting. Sustainable Energy Fuels 2023, 7 (1), 172–180. 10.1039/D2SE01295B.

[ref41] Xu Y.; Zhu K.; Yang X.; Zhu Y.; Jiang K.; Liu L.; Song H.; Chen Y.; Wang S.; Wang Z. Efficient application of new porous carbon nanoparticle composite polyaniline material in microbial fuel cells. Ind. Crops Prod. 2023, 192, 116130 10.1016/j.indcrop.2022.116130.

[ref42] Zheng W.; Sun Y.; Shu D.; Fan L.; Xu W.; Xu J. Compressible polyaniline-coated sodium alginate-cattail fiber foam for efficient and salt-resistant solar steam generation. J. Colloid Interface Sci. 2023, 645, 551–559. 10.1016/j.jcis.2023.04.182. 37163801

[ref43] Gao M.; Zhang G.; Zhang G.; Wang X.; Wang S.; Yang Y. The resistance to over-oxidation for polyaniline initiated by the resulting quinone-like molecules. Polym. Degrad. Stab. 2011, 96 (10), 1799–1804. 10.1016/j.polymdegradstab.2011.07.017.

[ref44] Kumar R.; Oves M.; Almeelbi T.; Al-Makishah N. H.; Barakat M. A. Hybrid chitosan/polyaniline-polypyrrole biomaterial for enhanced adsorption and antimicrobial activity. J. Colloid Interface Sci. 2017, 490, 488–496. 10.1016/j.jcis.2016.11.082. 27918986

[ref45] Zhu H.; Xie Y. Hydrogen-bonding interaction promoted supercapacitance of polylactic acid-graphene-microcrystalline cellulose/polyaniline nanofiber. Mater. Today Chem. 2023, 30, 101535 10.1016/j.mtchem.2023.101535.

[ref46] Wang K.; Song H.; Wang Z.; Liu L.; Li T.; Wang Y.; Han Y. Redox-active graphene/polypyrrole composite aerogel with high-performance capacitive behavior for flexible supercapacitor. Diamond Relat. Mater. 2023, 132, 109646 10.1016/j.diamond.2022.109646.

[ref47] Khan A.; Nawaz Bhatti H.; Tahira M.; Othman Alqahtani F.; Al-Fawzan F. F.; Alissa S. A.; Iqbal M. Na-alginate, polyaniline and polypyrrole composites with cellulosic biomass for the adsorptive removal of herbicide: Kinetics, equilibrium and thermodynamic studies. Arabian J. Chem. 2023, 16 (1), 104399 10.1016/j.arabjc.2022.104399.

[ref48] Ahmad S.; Khan I.; Husain A.; Khan A.; Asiri A. M. Electrical Conductivity Based Ammonia Sensing Properties of Polypyrrole/MoS2 Nanocomposite. Polymers 2020, 12 (12), 3047 10.3390/polym12123047. 33353209 PMC7767276

[ref49] Husain A.; Mahajan D. K. Effect of multi-walled carbon nanotubes on DC electrical conductivity and acetone vapour sensing properties of polypyrrole. Carbon Trends 2022, 9, 100193 10.1016/j.cartre.2022.100193.

[ref50] Han Y.; Wang T.; Li T.; Gao X.; Li W.; Zhang Z.; Wang Y.; Zhang X. Preparation and electrochemical performances of graphene/polypyrrole nanocomposite with anthraquinone-graphene oxide as active oxidant. Carbon 2017, 119, 111–118. 10.1016/j.carbon.2017.04.030.

[ref51] Hazarika J.; Kumar A. Controllable synthesis and characterization of polypyrrole nanoparticles in sodium dodecylsulphate (SDS) micellar solutions. Synth. Met. 2013, 175, 155–162. 10.1016/j.synthmet.2013.05.020.

[ref52] Gao Y.; Picot O. T.; Bilotti E.; Peijs T. Influence of filler size on the properties of poly(lactic acid) (PLA)/graphene nanoplatelet (GNP) nanocomposites. Eur. Polym. J. 2017, 86, 117–131. 10.1016/j.eurpolymj.2016.10.045.

[ref53] Armentano I.; Fortunati E.; Burgos N.; Dominici F.; Luzi F.; Fiori S.; Jimenez A.; Yoon K.; Ahn J.; Kang S.; Kenny J. M. Processing and characterization of plasticized PLA/PHB blends for biodegradable multiphase systems. eXPRESS Polym. Lett. 2015, 9 (7), 583–596. 10.3144/expresspolymlett.2015.55.

[ref54] Namhongsa M.; Daranarong D.; Sriyai M.; Molloy R.; Ross S.; Ross G. M.; Tuantranont A.; Tocharus J.; Sivasinprasasn S.; Topham P. D.; Tighe B.; Punyodom W. Surface-Modified Polypyrrole-Coated PLCL and PLGA Nerve Guide Conduits Fabricated by 3D Printing and Electrospinning. Biomacromolecules 2022, 23 (11), 4532–4546. 10.1021/acs.biomac.2c00626. 36169096

[ref55] da Silva A. M. G.; Barcelos K. d. A.; da Silva M. C.; Morelli C. L. Blend of recycled poly (ethylene terephthalate) and polycarbonate with polyaniline for antistatic packaging. Polym. Polym. Compos. 2020, 28 (5), 331–337. 10.1177/0967391119874994.

[ref56] Lapka T.; Vilčáková J.; Kopecký D.; Prokeš J.; Dendisová M.; Moučka R.; Sedlačík M.; Hassouna F. Flexible, ultrathin, and light films from one-dimensional nanostructures of polypyrrole and cellulose nanofibers for high-performance electromagnetic interference shielding. Carbohydr. Polym. 2023, 309, 120662 10.1016/j.carbpol.2023.120662. 36906374

[ref57] Qian J.; Xiao R.; Su F.; Guo M.; Liu D. 3D wet-spinning printing of wearable flexible electronic sensors of polypyrrole@polyvinyl formate. J. Ind. Eng. Chem. 2022, 111, 490–498. 10.1016/j.jiec.2022.04.030.

[ref58] Heo M.-S.; Kim T.-H.; Chang Y.-W.; Jang K. S. Near-Infrared Light-Responsive Shape Memory Polymer Fabricated from Reactive Melt Blending of Semicrystalline Maleated Polyolefin Elastomer and Polyaniline. Polymers 2021, 13 (22), 3984 10.3390/polym13223984. 34833283 PMC8618263

[ref59] Ilyas R. A.; Zuhri M. Y. M.; Aisyah H. A.; Asyraf M. R. M.; Hassan S. A.; Zainudin E. S.; Sapuan S. M.; Sharma S.; Bangar S. P.; Jumaidin R.; Nawab Y.; Faudzi A. A. M.; Abral H.; Asrofi M.; Syafri E.; Sari N. H. Natural Fiber-Reinforced Polylactic Acid, Polylactic Acid Blends and Their Composites for Advanced Applications. Polymers 2022, 14 (1), 202 10.3390/polym14010202. 35012228 PMC8747475

[ref60] Kalva S. N.; Ali F.; Velasquez C. A.; Koç M. 3D-Printable PLA/Mg Composite Filaments for Potential Bone Tissue Engineering Applications. Polymers 2023, 15 (11), 2572 10.3390/polym15112572. 37299370 PMC10255384

[ref61] Zhang Q.; Gao Y.; Luo B.; Cui Y.; Shu S.; Chen W.; Wang L. Effect of Styrene-Maleic Anhydride Copolymer on Properties of PBST/PLA Blends. Polymers 2023, 15 (4), 952 10.3390/polym15040952. 36850235 PMC9960150

[ref62] Zhang K.; Chen Z.; Smith L. M.; Hong G.; Song W.; Zhang S. Polypyrrole-modified bamboo fiber/polylactic acid with enhanced mechanical, the antistatic properties and thermal stability. Ind. Crops Prod. 2021, 162, 113227 10.1016/j.indcrop.2020.113227.

[ref63] Talebi A.; Labbaf S.; Karimzadeh F. A conductive film of chitosan-polycaprolcatone-polypyrrole with potential in heart patch application. Polym. Test. 2019, 75, 254–261. 10.1016/j.polymertesting.2019.02.029.

[ref64] Roca F. G.; García-Bernabé A.; Compañ Moreno V.; Martínez-Ramos C.; Monleón Pradas M. Solid Polymer Electrolytes Based on Polylactic Acid Nanofiber Mats Coated with Polypyrrole. Macromol. Mater. Eng. 2020, 306 (2), 2000584 10.1002/mame.202000584.

[ref65] Jafari A.; Fakhri V.; Kamrani S.; Reza Ghaffarian Anbaran S.; Su C.-H.; Goodarzi V.; Pirouzfar V.; Ali Khonakdar H. Development of Flexible Nanocomposites Based on Poly(ε-caprolactone) for Tissue Engineering Application: The Contributing Role of Poly(glycerol succinic acid) and Polypyrrole. Eur. Polym. J. 2022, 164, 110984 10.1016/j.eurpolymj.2021.110984.

[ref66] He Z.; Lin H.; Zhang X.; Chen Y.; Bai W.; Lin Y.; Jian R.; Xu Y. Self-healing epoxy composite coating based on polypyrrole@MOF nanoparticles for the long-efficiency corrosion protection on steels. Colloids Surf., A 2023, 657, 130601 10.1016/j.colsurfa.2022.130601.

[ref67] Yang Y.; Liu Y.; Zhao X. Preparation and characterization of an electromagnetic composite polypyrrole/polyethylene short filament geotextile. Text. Res. J. 2021, 92 (7–8), 1333–1343. 10.1177/00405175211053396.

[ref68] Parangusan H.; Bhadra J.; Ahmad Z.; Mallick S.; Touati F.; Al-Thani N. Humidity sensor based on poly(lactic acid)/PANI-ZnO composite electrospun fibers. RSC Adv. 2021, 11, 28735–28743. 10.1039/D1RA02842A. 35478584 PMC9038121

[ref69] Doumeng M.; Makhlouf L.; Berthet F.; Marsan O.; Delbé K.; Denape J.; Chabert F. A comparative study of the crystallinity of polyetheretherketone by using density, DSC, XRD, and Raman spectroscopy techniques. Polym. Test. 2021, 93, 106878 10.1016/j.polymertesting.2020.106878.

[ref70] Du M.; Guo C.; Cai Y.; Liu J.; Wei Q.; Li L. Multifunctional shape-stabilized phase change composites based upon multi-walled carbon nanotubes and polypyrrole decorated melamine foam for light/electric-to-thermal energy conversion and storage. J. Energy Storage 2021, 43, 103187 10.1016/j.est.2021.103187.

[ref71] Zhuo H.; Hu Y.; Chen Z.; Zhong L. Cellulose carbon aerogel/PPy composites for high-performance supercapacitor. Carbohydr. Polym. 2019, 215, 322–329. 10.1016/j.carbpol.2019.03.101. 30981361

[ref72] Mao W.; Zhang Y.; Luo J.; Chen L.; Guan Y. Novel co-polymerization of polypyrrole/polyaniline on ferrate modified biochar composites for the efficient adsorption of hexavalent chromium in water. Chemosphere 2022, 303 (Part 3), 135254 10.1016/j.chemosphere.2022.135254. 35690169

[ref73] Xu Z.; Zheng E.; Xiao Z.; Shao H.; Liu Y.; Wang J. Photo-Initiated in situ synthesis of polypyrrole Fe-Coated porous silicon microspheres for High-performance Lithium-ion battery anodes. Chem. Eng. J. 2023, 459, 141543 10.1016/j.cej.2023.141543.

[ref74] Wang J.; Zhang K.; Zhao L. Sono-assisted synthesis of nanostructured polyaniline for adsorption of aqueous Cr (VI): Effect of protonic acids. Chem. Eng. J. 2014, 239, 123–131. 10.1016/j.cej.2013.11.006.

[ref75] Tabačiarová J.; Mičušík M.; Fedorko P.; Omastová M. Study of polypyrrole aging by XPS, FTIR and conductivity measurements. Polym. Degrad. Stab. 2015, 120, 392–401. 10.1016/j.polymdegradstab.2015.07.021.

[ref76] Raghunathan S. P.; Narayanan S.; Poulose A. C.; Joseph R. Flexible regenerated cellulose/polypyrrole composite films with enhanced dielectric properties. Carbohydr. Polym. 2017, 157, 1024–1032. 10.1016/j.carbpol.2016.10.065. 27987802

